# Salmonella Septic Hip Arthritis in Immunocompetent Children: Three Case Reports and Literature Review

**DOI:** 10.7759/cureus.27701

**Published:** 2022-08-05

**Authors:** Maria Tirta, Dimitris Ampelas, Panagiotis Tsintavis, Anastasia Pilichou, Panayotis Krallis

**Affiliations:** 1 Orthopaedics and Traumatology, Agia Sofia Children's Hospital, Athens, GRC; 2 Epidemiology and Public Health, National and Kapodistrian University of Athens, Athens, GRC; 3 Orthopaedics and Traumatology, Livadeia General Hospital, Livadeia, GRC

**Keywords:** infant, immunocompetent children, case report, septic arthritis, salmonella

## Abstract

Septic arthritis is an orthopaedic emergency, especially considering the pediatric population. Salmonella is a less common aetiologic factor for septic arthritis compared with other Gram-negative bacteria. Its isolation in immunocompetent children and infants is quite rare, with an estimated incidence of 0.1% to 0.2% of septic arthritis cases among children.

We report three rare cases of hip septic arthritis with Salmonella as a confirmed pathogen in immunocompetent children, with two of them being infants. The main symptoms that occurred in all three cases were fever, hip pain/no bearing, and diarrhoea, as well as elevated levels of WBC, C-reactive protein and ESR. The duration of their hospitalization was 20, 26 and 52 days. One case was treated only with antibiotics, while the other two with surgical drainage with/without arthrotomy. All cases had a follow-up of at least one year, with only one of the infants having the femoral head in a subluxated position and early signs of osteonecrosis. Fluoroscopy-assisted closed reduction had to be performed and maintained by hip spica. A new femoral epiphysis was formed at the time of the last follow-up 2.5 years later.

Our case series highlights the possibility of Salmonella typhi septic hip arthritis in immunocompetent individuals. Therefore, Salmonella species must always be kept in mind during the differential diagnosis of septic arthritis in a clinically relevant setting.

## Introduction

Septic arthritis is an important musculoskeletal infection in children and a true orthopaedic emergency [1]. Any delay in diagnosis and treatment may result in irreversible damage to the joint. The knee and hip are the most commonly affected sites followed by the shoulder and other joints [2]. Before the widespread use of vaccination against Haemophilus Influenza type B, the latter was considered the main cause of septic arthritis [3]. Nowadays, *Staphylococcus* *aureus* and *Streptococci A* are responsible for the majority of the confirmed cases [4]. Salmonella sub-species are often isolated in elderly patients, immunocompromised patients, or patients with some sort of haemoglobinopathy [5,6]. Septic arthritis due to Salmonella species in immunocompetent children is a rare entity [7]. In this report, we present three rare cases of Salmonella septic arthritis of the hip in otherwise healthy children. This report has been prepared according to the SCARE guidelines [8].

## Case presentation

Case 1

A 10-year-old boy presented at our hospital's emergency department with symptoms of septic arthritis of the left hip. The patient had a five-day history of vomiting and diarrhoea, abdominal pain, fever (up to 39 ^o^C) and a gradual inability to bear weight on his left side. The patient had no risk factors and no previous history of severe illness. There was no history or evidence of trauma in the affected limb. At the time of the orthopaedic examination, the left hip was in 30^o^ flexion, slight abduction, and external rotation. Blood tests showed leukocytosis (WBC 10.54 x10^3^/μL) and a raised C-reactive protein (CRP 114mg/L). There was no sign of concurrent infection in other sites of the body.

Joint aspiration, drainage and washout were performed, and the patient received empirical treatment with I.V. Ceftriaxone and Amikacin for 10 days. Direct microscopic examination and Gram stain of the synovial fluid revealed the presence of Gram-negative bacteria. Synovial fluid culture developed Salmonella group A (paratyphi) susceptible to Ceftriaxone and Cefixime. The family of the patient was investigated via coproculture, but no *Salmonella *spp was discovered. Following surgical drainage, the patient’s symptoms improved dramatically. Based on the sensitivity results Amikacin was discontinued and Ceftriaxone was continued as monotherapy for a total of 15 days. On discharge, the CRP had declined to 1.36mg/L and the patient was prescribed orally Cefixime for another four weeks, to complete a six-week course, and started to bear weight after two weeks from the discharge with the use of crutches for 10 days. One year later, during the last follow-up visit, the affected joint was totally functional (flexion 120^o^, hyperextension 10^o^, abduction 45^o^, internal and external rotation 45^o^) and there was no sign of avascular necrosis of the femoral head.

Case 2

A nine-month-old girl was transferred to our department from a regional hospital with persistent septic arthritis of the left hip that did not respond to intravenous antibiotic treatment. The patient had a history of minor episodes of diarrhoea about a month before the onset of arthritis, but no signs or symptoms of systemic disease. She had no significant medical illness since birth, birth age of 38 weeks, no complications during pregnancy or labour and her mother was healthy without known immunodeficiency. At the regional hospital, they performed an urgent arthrotomy and surgical drainage. Postoperatively the patient was given I.V. ceftriaxone and her leg was kept in traction. Synovial fluid and blood cultures indicated a Ceftriaxone-sensitive Salmonella *enteritidis* as the main culprit. The inflammatory markers were still raised after a month of antibiotic treatment (ERS 65mm/hr, CRP 31mg/L) requiring a second surgical debridement.

**Figure 1:**
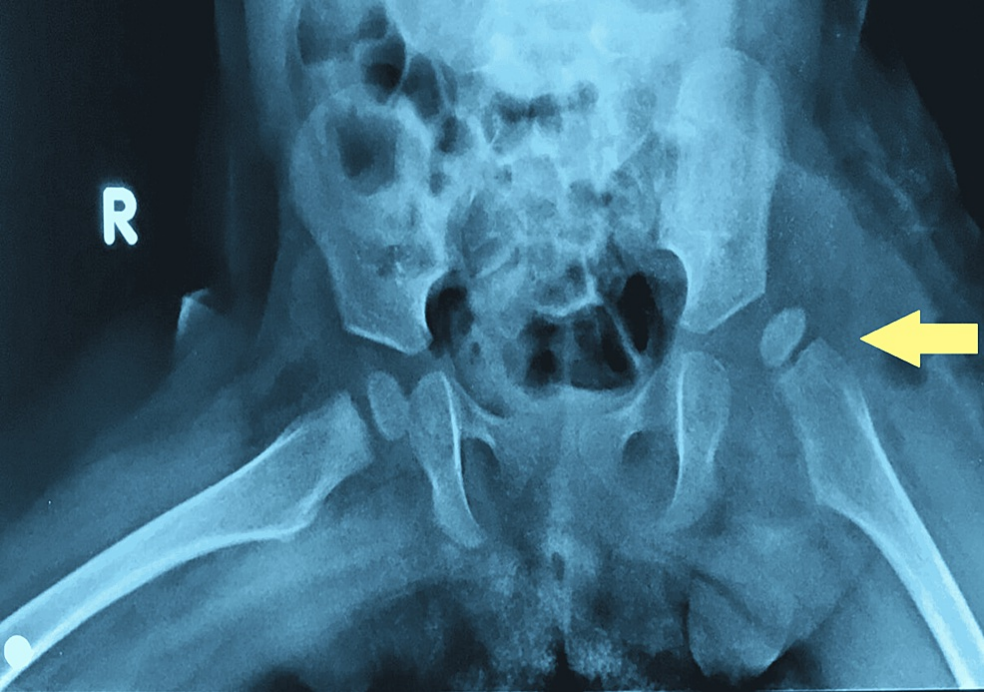
Subluxation of the left femoral head in a nine-month girl with persistent septic arthritis of the hip. The yellow arrow shows the subluxation of the left femoral head. Fluoroscopy-assisted closed reduction was performed and maintained with a hip spica cast.

**Figure 2:**
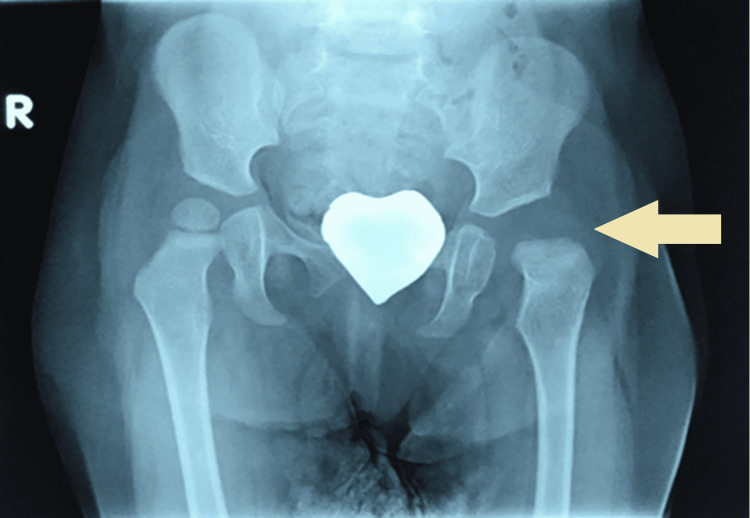
Vanishing of the left femoral head due to osteonecrosis caused by the infection. The yellow arrow shows that the epiphysis of the femoral head had vanished due to the ongoing osteonecrosis.

**Figure 3:**
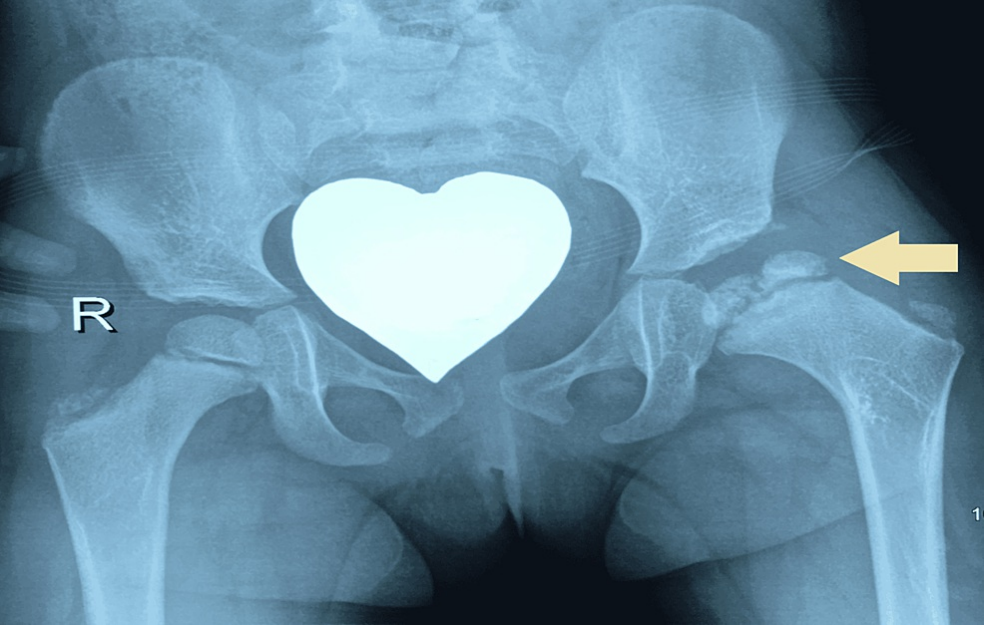
Two years after the initial infection there is a significant remodelling of the femoral head. The yellow arrow the remodeling of the femoral head after two years. The patient has no symptoms and is able to bear weight.

Imaging with plain x-rays was performed in our department and showed that the head of the left femur was in a subluxated position (Figure 1), while MRI showed early signs of osteonecrosis of the femoral head. Fluoroscopy-assisted closed reduction had to be performed and maintained by hip spica. Ceftriaxone IV treatment continued until the closed reduction, after which Ciprofloxacin was added to the treatment, and after a three-week course of IV antibiotics, the patient was discharged having negative inflammation markers. Two months later hip spica was removed. There was no sign of inflammation and serological markers were within normal values, but the epiphysis of the femoral head had vanished due to the ongoing osteonecrosis (Figure 2). In the follow-up visits, the patient showed significant remodelling potential on par with her young age and was able to bear weight and started walking six months later. A new femoral epiphysis had been formed at the time of her last follow-up 2.5 years later (Figure 3) and the affected joint was totally functional (flexion 115^o^, hyperextension 10^o^, abduction 45^o^, internal and external rotation 45^o^).

Case 3

A nine-month-old boy was transferred to our department from a regional hospital with the diagnosis of salmonellosis based on blood and faecal culture results. The boy's past medical history was free from any systemic or autoimmune disease, had no significant medical illness since birth, birth age of 40 weeks, no complications during pregnancy or labour and his mother was healthy without known immunodeficiency. The initial symptoms - a month before the child’s admission to hospital - were multiple episodes of diarrhoea (6-8/day) which resolved one week later without treatment. The boy was free of symptoms for about a week before the onset of prolonged fever for five days. In addition, the child couldn’t bear weight on his left side when raised on a standing position and had his left hip held in abduction and external rotation when laid down and was admitted to the regional hospital. Upon admission, the blood tests revealed leukocytosis (13.66 x 10^3^/μL) and high inflammatory markers (ESR 75mm/hr, CRP 51mg/L) and clinical examination showed limited range of motion (ROM) on his left hip. Cefotaxime, Clindamycin, and Ampicillin I.V. were administered, and the initial x-ray evaluation was normal. Blood and faecal cultures identified *Salmonella enteritidis* as the cause of infection and the antibiotic regimen was modified accordingly. Nevertheless, despite the intravenous administration, the episodes of fever and diarrhoea as well as the limited ROM of the left hip persisted for one more week. An MRI of the pelvis and a three-phase bone scan revealed signs of infection of the left hip, while an ultrasound of the hip, which was performed a week later, highlighted the presence of fluid in the articular bursa.

It was decided that the boy should be transferred to a tertiary centre, where the antibiotic regimen was modified to exclude Ampicillin. The inflammatory markers remained high (CRP 30.2mg/L, ESR 125mm/hr). During the next days, the range of ROM of the left hip gradually improved and a week later decrease in the inflammation markers was shown (CRP 3.03mg/L, ESR 80mm/hr). One week later, on discharge, the boy had gained full ROM of his left hip, with a further decrease of the inflammatory markers (CRP 1.71mg/L, ESR 50mm/hr) and no episodes of fever. During the follow-up visits (six months, one year and two years), the boy’s hip was fully functional (flexion 120^o^, hyperextension 10^o^, abduction 45^o^, internal and external rotation 45^o^) and the x-rays revealed no signs of osteonecrosis on the femoral head.

Table 1 summarizes the main characteristics of the three cases. Table 2 presents the culture reports of the three patients showing organisms isolated, antibiotic sensitivities, inflammatory markers and treatment.

**Table 1:** Characteristics of the three cases with Salmonella septic hip arthritis Range of inflammatory markers' normal values for our hospital's lab: WBC - 3.6-9.5x10^3^/μL, CRP - 1-10mg/L, ESR - 3-13mm/hr

	Case 1	Case 2	Case 3
Sex	Male	Female	Male
Age	10 years	9 months	10 months
Hip	Left	Left	Left
Symptoms			
Fever	Yes	Yes	Yes
Hip Pain- No bearing	Yes	Yes	Yes
Vomiting	Yes	No	No
Abdominal pain	Yes	No	No
Diarrhoea	Yes	Yes	Yes
Blood Tests			
White Blood Cells (WBC)	10.54 x10^3^/μL	N/A	13.66 x 10^3^/μL
C-Reactive Protein (CRP)	114 mg/L	50 mg/L	51 mg/L
Erythrocyte Sedimentation Rate (ESR)	50 mm/hr	65 mm/hr	75mm/hr

**Table 2:** Culture reports of three patients showing organisms isolated, antibiotic sensitivities, inflammatory markers and treatment

	Case 1	Case 2	Case 3
Organism	Salmonella group A (paratyphi)	Salmonella enteritidis	Salmonella enteritidis
Treatment	Surgical drainage	Arthrotomy and Surgical drainage (2), Fluoroscopy-assisted closed reduction & hip spica	Antibiotics
Susceptible to	Ceftriaxone and Cefixime	Ceftriaxone	Cefotaxime and Clindamycin
Antibiotic Therapy	Ceftriaxone IV	Ceftriaxone IV and Ciprofloxacin added	Cefotaxime, Clindamycin and Ampicillin, then without Ampicilin
C-reactive protein (last)	1.36 mg/L	< 2 mg/L	1.71 mg/L
Hospitalization	26 days	52 days	20 days
Follow-up- Outcome	Joint fully functional, x-rays no signs of osteonecrosis of femoral head	Early signs of osteonecrosis of femoral head	Joint fully functional, x-rays no signs of osteonecrosis of femoral head
Final	1 year	2.5 years	2 years

## Discussion

Septic arthritis caused by Salmonella is very rare and occurs in approximately 1% of all cases [9], while an early diagnosis of septic arthritis in children is very important because delayed or inadequate treatment carries a risk of permanent disability. Its presence in immunodeficient children or children suffering from an underlying chronic disease is well documented in the literature [10-12]. However, septic arthritis of the hip in otherwise healthy children is infrequent and very few cases have been reported so far.

We performed a literature review to identify all the cases of septic arthritis caused by Salmonella in previously healthy children. PubMed was searched using the search terms “typhoid” or “Salmonella” and “septic arthritis” or “joint infection”, restricting our search to include only those reports of cases in immunocompetent children, up to 16 years of age within the last 20 years, written in the English language. Nine publications were identified, with a total of 13 cases, the details of which are shown in Table 3 [9,13-20]. The age range was from nine months to 16 years old. The hip was the affected joint in most cases, with two cases of shoulder and one knee. Management was typically with a combination of surgical drainage by arthrotomy and prolonged antibiotics. Balakumar et al. highlight that it is difficult to clinically differentiate Salmonella septic arthritis from other usual pathogens as we have to rely on the culture reports, but those who fail to respond to standard antibiotics for the common pathogens should be investigated for other unusual ones [18], as happened in our cases.

**Table 3:** Articles reporting cases of Salmonella septic arthritis in immunocompetent children during the past 20 years A, ampicillin; Am, Amoxycillin; Amk, amikacin; AmoCl, Amoxicillin clavulanic acid; C, chloramphenicol; Cef, cefixime; Cro, ceftriaxone; Cz, Ceftazidime; Cfo, Cefotaxime; Ci, Clindamycin; Cip, ciprofloxacin; Cipi, intermediate susceptibility to ciprofloxacin; Co, Cotrimaxazole; Cu, Cefuraxime; Ge, Gentamycin; Me, Meropenem; Na, nalidixic acid; Ofl, ofloxacin; Su, sulphamethoxazole; Te, Tetracycline; Tm, trimethoprim; IV, intravenous; PO, oral; yr, years; mo, months; wks, weeks.

Author	Year	Country	Age	Joint Affected	Resistance/ Sensitive Antibiotics	Surgery	Treatment	Outcome	Duration Follow-up
Chiu [9]	2001	Taiwan	2.5 yr	Hip	Not reported	Arthotomy	4 weeks Cro	Recovered	Not reported
Agnihotri [13]	2005	India	7 yr	Hip	Fully susceptible	Arthotomy	IV Cip for 5 days, PO Cip for 10 days	Recovered	15 days
Faseela TS [14]	2010	India	16 yr	Hip	Sensitive: Am C Co Cip Cef Cro Cfo Ofl	No surgery	Cip (time not reported)	Recovered	Not reported
Halim [15]	2011	Malaysia	4 yr	Hip	Sensitive: AmoCl Cro A	Arthotomy, wash out	1 week IV AmoCl , PO AmoCl for 6 weeks	Recovered	1 yr
Mahajan RK [16]	2012	India	3 yr	Hip	Resistance: A Na	Arthotomy	2 weeks IV Cro/Ofl	Recovered	Not reported
			5 yr	Hip	Resistance: A Na	Aspiration; subsequent arthrotomy & washout	2 weeks IV Cro/Ofl	Recovered	3 wks
			6 yr	Hip	Resistance: A Na	Arthotomy	2 weeks IV Cro/Ofl	Recovered	Not reported
Pocock [17]	2014	Cambodia	12 yr	Hip	Resistance: C A Tm Su Na Cipi	Drainage & washout	2 weeks IV Cro, 4 weeks PO Azm	Recovered	6 wks
Balakumar [18]	2017	India	9 mo	Shoulder	Sensitive: A Na C Cip Co	Arthotomy & Decompression	4 weeks Cip	Recovered	6 mo
			18 mo	Shoulder	Sensitive: A Co Cro C Cipi	Arthotomy	2 weeks IV Cro, 4 weeks PO Cu	Recovered	10 wks
			11 yr	Hip	Sensitive: A Co Cro C Cipi	Aspiration; subsequent arthrotomy & washout	2 weeks IV Cip/Cro,4 weeks PO Cip/Cef	Recovered	Not reported
Tassinari [19]	2018	Brasil	11 yr	Hip	Sensitive: A Cro Su Tm	Arthrotomy & drainage	4 weeks IV Cro	Recovered	Not reported
Kurniawan [20]	2021	Indonesia	2 yr	Knee	Sensitive: C Co Ge Te Amk A Cfo AmoCl Cro Me Cz	Aspiration; subsequent arthrotomy & debridement	1 week IV AmoCl, 1 week PO AmoCl	Recovered	5 mo
Current	2022	Greece	10 yr	Hip	Sensitive: Cro Cef	Drainage & washout	2 weeks IV Cro, 4 weeks PO Cef	Recovered	1 yr
			9 mo	Hip	Sensitive: Cro	Arthotomy, Drainage & washout	3 weeks IV Cro/Cip	Osteonecrosis of the femoral head, Recovered	2.5 yr
			10 mo	Hip	Sensitive: Cfo Ci	No surgery	3 weeks IV Ci/Cu	Recovered	2 yr

Our report is the only one that describes the treatment of an infant with early signs of osteonecrosis of the femoral head due to Salmonella septic arthritis. It is of great importance that in neonates and infants septic arthritis is characterized by atypical clinical picture, often causing delayed diagnosis, and in the initial phases of the disease ultrasonographic findings are of greater use compared to radiological imaging, due to relatively late appearance of radiological signs of disease. With the current case report, we highlight that among infants' joint effusion can result in the dislocation of the hip joint and may lead to instability requiring hip spica casting.

Also, we have the longest follow-up in all the three cases (1, 2.5 and 2 years, respectively) compared to the other reports. This is another strength of our study, since the complications may develop slowly, and a long follow-up of one to two years may be required to detect all possible sequelae, especially concerning infants.

This study is not without limitations. Being a retrospective study, it has the inherent limitations of such studies and inference needs to be drawn with caution.

## Conclusions

Septic arthritis due to Salmonella is a rarity but still a reality at a young age, even for immunocompetent individuals. It can be resistant to treatment with long-term sequelae for the patient. Immediate surgical drainage providing synovial fluid cultures is the cornerstone for both diagnosis and treatment. The importance of appropriate antibiotic therapy and a long follow-up cannot be overstated.
